# Coexistence of non-functional ectopic thyroid tissue and a normal thyroid: A case report

**DOI:** 10.3892/etm.2013.1244

**Published:** 2013-08-02

**Authors:** WEI ZHENG, JIAN TAN, TONG LIU

**Affiliations:** 1 Departments of Nuclear Medicine, Tianjin Medical University General Hospital, Heping, Tianjin 300052, P.R. China; 2 General Surgery, Tianjin Medical University General Hospital, Heping, Tianjin 300052, P.R. China

**Keywords:** ectopic, thyroid, cervical mass, non-functional, histology

## Abstract

The aim of this study was to report a rare case of non-functional ectopic thyroid tissue in the neck with a coexisting normal thyroid. A 36-year-old female presented with a mass in the anterior neck. The thyroid function of the patient was normal. Ultrasonography and computed tomography (CT) revealed a normal thyroid gland and a solid mass in the middle lower neck. Scintigraphy showed a normal thyroid and the mass did show any uptake of ^99m^TcO_4^−^_. The patient underwent en bloc resection. During surgery, the mass was observed to be well encapsulated and completely separated from the thyroid gland. Histology revealed it as ectopic thyroid tissue. The patient had an uneventful recovery.

## Introduction

Ectopic thyroid tissue, defined as thyroid tissue not located anterior-laterally to the second and fourth tracheal cartilages, is rare. It is a congenital disease caused by the abnormal migration of thyroid tissue in the embryonic stage (1). In the majority of cases, ectopic thyroid is located in the midline, between the foramen caecum and the normal location of the thyroid gland, and most often it is located in the base of the tongue (2,3). Patients usually present with symptoms, including a palpable but asymptomatic neck mass, dysphagia, dysphonia or dyspnea, according to the location and size of the mass. In the majority of cases, ectopic thyroid is the only thyroid tissue present (1). The current report describes a rare case: a 36-year-old female patient presented a non-functional ectopic thyroid tissue in the lower neck with a coexisting normal thyroid, which is infrequent in the clinic. The present study was approved by the ethics committee of Tianjin Medical University General Hospital (Tianjin, China) and adhered to the tenets of the Declaration of Helsinki. In addition, the written informed consent was obtained from the patient.

## Case report

**Figure 1.**
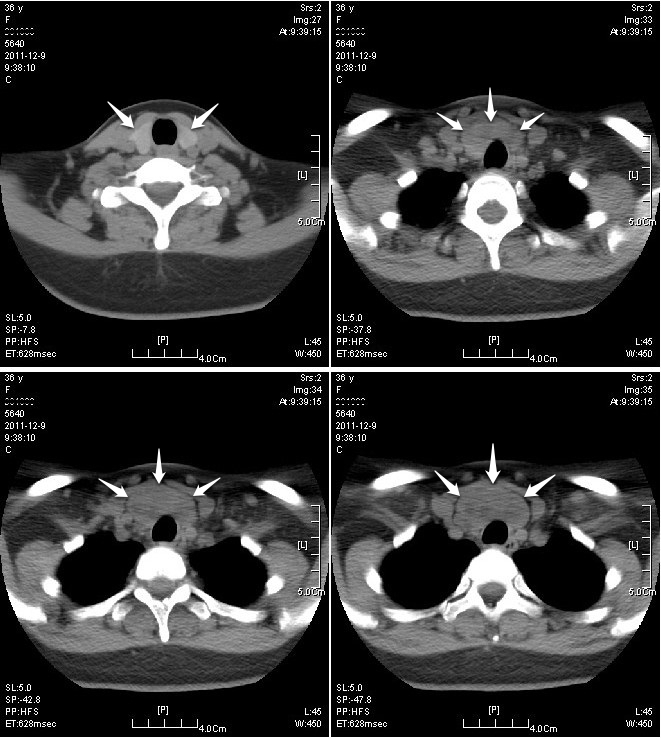
Computed tomography (CT) scans of the patient’s neck revealed a well-defined mass below the thyroid gland on the border between the cervix and the thorax.

**Figure 2.**
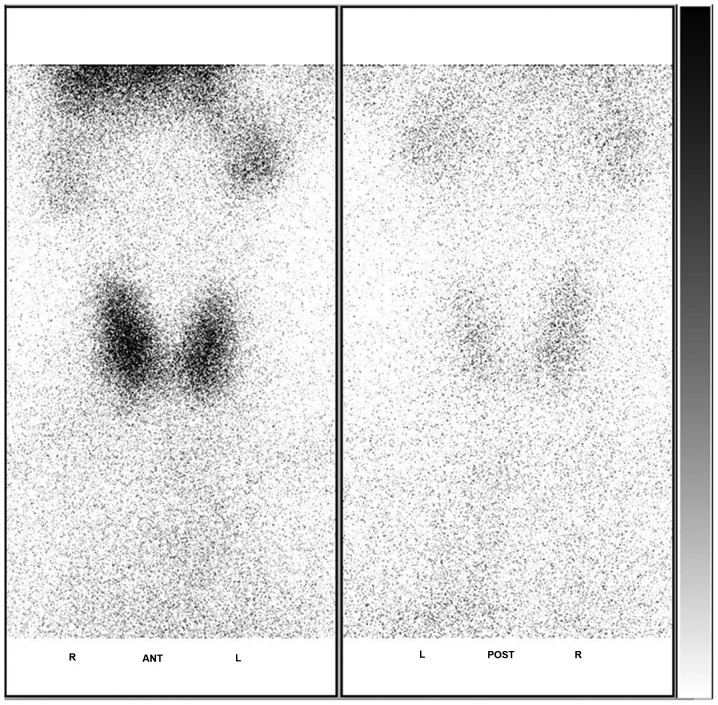
In ^99m^TcO_4^−^_ scintigraphy of the patient’s neck, only the normal thyroid gland was shown and the cervical mass did not take up the radionuclide.

A 36-year-old female presented with a recent onset of a painless mass in the middle of the lower neck. The patient was asymptomatic and there was nothing significant in the medical history. Physical examination revealed a ∼4 cm immobile, non-tender mass in the patient’s lower neck. Thyroid function tests, as well as the thyroglobulin (Tg) and thyroglobulin antibody (TgAb) levels of the patient were normal: free triiodothyronine (FT_3_), 4.65 pmol/l (normal range, 3.5–6.5 pmol/l); free thyroxine (FT_4_), 18.52 pmol/l (normal range, 11.5–23.5 pmol/l); sensitive thyroid stimulating hormone (sTSH), 1.38 *μ*IU/ml (normal range, 0.3–5.0 *μ*IU/ml); Tg, <0.20 ng/ml (normal range, 0–55 ng/ml); and TgAb, 26 IU/ml (normal range, 0–40 IU/ml). Thyroid hormones and TSH were determined with an immunofluorometric assay. Tg and TgAb were measured with a radioimmunoassay. Blood serum calcitonin, calcium and parathyroid hormone (PTH) levels were normal. A neck ultrasound revealed a normal thyroid gland and a solid mass of heterogeneous echotexture in the middle lower cervical area, measuring 5.1 × 2.8 cm. The patient refused a fine needle aspiration (FNA) biopsy. A computed tomography (CT) scan of the neck revealed a well-defined mass below the thyroid gland, at the border of the cervical region and the thorax (Fig. 1). The mass suppressed the trachea; however, it was not causing narrowing of the trachea. ^99m^TcO_4^−^_ scintigraphy of the patient’s neck revealed only the normal thyroid gland; the cervical mass did not show any uptake of ^99m^TcO_4^−^_ (Fig. 2).

**Figure 3.**
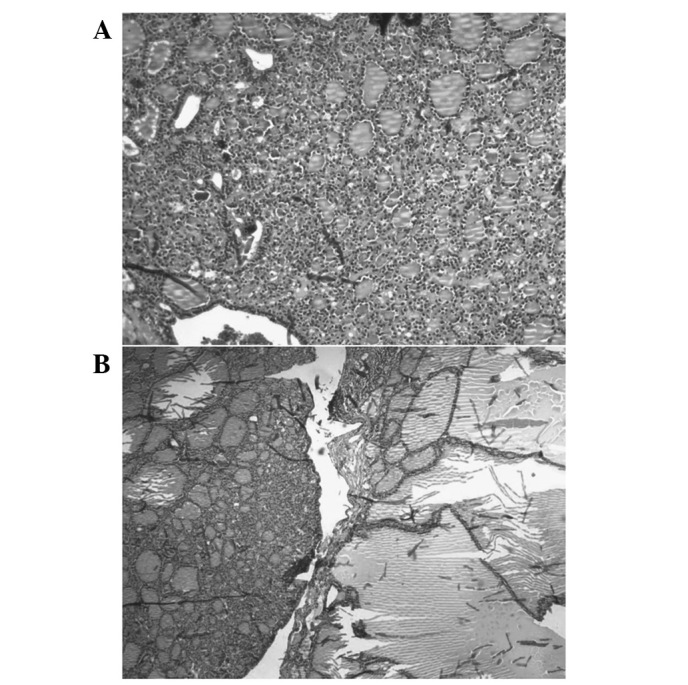
Histology revealed ectopic thyroid tissue with adenomatous nodules. (A) magnification, ×100; (B) magnification, ×40. The tissues were stained with hematoxylin and eosin.

The patient underwent en bloc resection, in which the cervical mass was identified as a separate structure from the thyroid gland. The patient had an uneventful postoperative recovery. Histological examination revealed that it showed large follicular cells of thyroid tissue distended with colloid material which confirms the characteristic of adenomatous hyperplasia (Fig. 3). Postoperatively the patient was euthyroid and had normal calcium levels.

## Discussion

Ectopic thyroid tissue, defined as thyroid tissue not located anterior-laterally to the second and fourth tracheal cartilages, is rare. It was first described by Hickman in 1869 in a newborn baby who suffocated 16 h after birth due to a lingual thyroid causing upper airway obstruction (4,5,6). It is a congenital disease caused by abnormal migration of thyroid tissue in the embryonic stage. During embryogenesis, the descent of the thyroid does not proceed normally, leading to various possible anomalous locations of the gland. According to the timing of the embryonic development, thyroid descent may stop at various sites, from the base of the tongue to any site of the thyroglossal duct (3). In the majority of cases it is located in the midline, between the foramen caecum and the proper location of the thyroid gland, and most often it is located in the base of the tongue (2,3). In the current study, a rare case of a non-functioning ectopic thyroid in the lower cervical area in a female patient with a normal thyroid gland is presented.

Lingual thyroid is the most common form of ectopic thyroid (7), which may cause dysphonia (8). Extralingual thyroid tissue is commonly located in the anterior cervical area, along the path of the thyroglossal duct (3). In the patient in the present study it was located underneath the thyroid in the lower neck, which is a rare location. Other rare locations of ectopic thyroid include the submandibular region (9), parotid salivary gland (10), trachea (11), lateral to the carotid arteries and jugular veins (7), mediastinum (12), heart (13), lung (14), duodenum (15), adrenal gland (16) and uterus (17).

The exact incidence of ectopic thyroid is unknown. Post-mortem studies suggest that asymptomatic thyroid tissue may be located along the path of the thyroglossal duct in as many as 7–10% of adults (18). Ectopic thyroid tissue may be the only functioning tissue (3,19,20) or may coexist with a normal thyroid gland (9,21,22), as in the present case.

Radionuclide imaging (RI) is considered the definitive diagnostic test method for detecting ectopic thyroid tissue. In the present case, no uptake of radiotracer was observed in the ectopic thyroid. In a previous study, the findings on color Doppler ultrasonography (CDU), gray-scale ultrasonography (GSU) and magnetic resonance imaging (MRI) were compared with those of RI and the sensitivities for detecting ectopic thyroid were calculated (23). In the patients with ectopic thyroid, the sensitivity of CDU, GSU and MRI for detecting ectopic thyroid was 90, 70 and 70%, respectively. CDU is superior to GSU and MRI for detecting ectopic thyroid.

Ectopic thyroid tissue may undergo the same pathological changes as the eutopic thyroid gland, including thyrotoxicosis (19), and may be benign or malignant (11,12,24). Malignant transformation of ectopic thyroid tissue is extremely uncommon. If, however, thyroid tissue is located in the lateral cervical lymph nodes, metastasis of a malignant thyroid tumor should be excluded (25).

Ectopic thyroid tissue poses difficult diagnostic and management challenges. The treatment of ectopic thyroid tissue depends on factors such as mass size, local symptoms, the age of the patient, the functional status of the thyroid gland and complications, including ulceration, hemorrhage and neoplasia (26,27). In order to prevent misdiagnosis and mismanagement, patients with a cervical mass in the anterior midline should be subjected to history screening, physical examination, cervical CDU, RI, thyroid function examinations (TSH, FT_3_ and FT_4_), FNA biopsy and histological examinations during surgery (23).
